# Would You Sacrifice Your Privacy to Protect Public Health? Prosocial Responsibility in a Pandemic Paves the Way for Digital Surveillance

**DOI:** 10.3389/fpsyg.2020.578618

**Published:** 2020-09-18

**Authors:** Michail D. Kokkoris, Bernadette Kamleitner

**Affiliations:** ^1^Marketing Department, Vrije Universiteit Amsterdam, Amsterdam, Netherlands; ^2^Marketing Department, Institute for Marketing and Consumer Research, WU Vienna University of Economics and Business, Vienna, Austria

**Keywords:** responsibility, prosocial behavior, digital surveillance, privacy, civil rights, freedom, location tracking, COVID-19 pandemic

## Abstract

Digital surveillance methods, such as location tracking apps on smartphones, have been implemented in many countries during the COVID-19 pandemic, but not much is known about predictors of their acceptance. Could it be that prosocial responsibility, to which authorities appealed in order to enhance compliance with quarantine measures, also increases acceptance of digital surveillance and restrictions of privacy? In their fight against the COVID-19 pandemic, governments around the world communicated that self-isolation and social distancing measures are every citizen’s duty in order to protect the health not only of oneself but also of vulnerable others. We suggest that prosocial responsibility besides motivating people to comply with anti-pandemic measures also undermines people’s valuation of privacy. In an online research conducted with US participants, we examined correlates of people’s willingness to sacrifice individual rights and succumb to surveillance with a particular focus on prosocial responsibility. First, replicating prior research, we found that perceived prosocial responsibility was a powerful predictor of compliance with self-isolation and social distancing measures. Second, going beyond prior research, we found that perceived prosocial responsibility also predicted willingness to accept restrictions of individual rights and privacy, as well as to accept digital surveillance for the sake of public health. While we identify a range of additional predictors, the effects of prosocial responsibility hold after controlling for alternative processes, such as perceived self-risk, impact of the pandemic on oneself, or personal value of freedom. These findings suggest that prosocial responsibility may act as a Trojan horse for privacy compromises.

## Introduction

In response to the COVID-19 pandemic, governments around the world besides appealing to people to comply with self-isolation and social distancing recommendations have also resorted to digital surveillance measures (Calvo et al., 2020). One of the most common forms of surveillance implemented is the use of smartphone location data (Amit et al., 2020; Heaven, 2020, March 17). For example, Israel has been using a technology originally developed for counterterrorism purposes to track the mobile phones of civilians in order to contain the spread of the virus (Livni, 2020, March 17). China has been tracking citizens in many cities through a smartphone app that assigns a green, yellow, or red color code as indication of one’s health status (Mozur et al., 2020, March 1). Even in privacy-conscious Europe, Austrian health authorities encouraged citizens to download a contact-tracing app developed for the pandemic by the Austrian Red Cross (Birnbaum and Spolar, 2020, April 18). Although these measures have been imposed for the protection of public health, they have stirred controversy due to potential threats to personal privacy and civil rights (Abbas et al., 2020; Calvo et al., 2020; Roth et al., 2020; Singer and Sang-Hun, 2020, April 17). Essentially, their implementation may result in the protection of public health at the price of a loss of individual freedoms.

In this research, we explore factors that make people accept such losses of individual freedoms. In particular, we focus on perceptions of prosocial responsibility as a factor that makes people willing to pay that price in a pandemic and accept an increase in digital surveillance. In the context of this research, we define prosocial responsibility as a state of heightened awareness that one’s behavior has consequences for others coupled with concerns about their well-being. In the COVID-19 pandemic, authorities have extensively appealed to prosocial responsibility as a way to motivate people to adhere to self-isolation and social distancing measures. Compliance with these measures is crucial in the fight of the pandemic. Literature shows that feeling responsible for others can have a large impact on people’s motivation and behavior. For example, consumers are willing to incur costs to buy products if they believe that these have a positive impact on society (Small and Cryder, 2016), or taxpayers support taxation if they recognize that their tax contributions help fellow citizens (Thornton et al., 2019). Research in ethical decision-making suggests that people do not want others to think about them that they are behaving selfishly; instead, they enjoy reputational benefits, such as respect and admiration, if they behave in line with what is considered normatively ‘good’ (Van Bavel et al., 2020).

More specific to the topic of the present investigation, the COVID-19 pandemic, a recent review of 3,166 papers on the psychological impact of quarantine demonstrated the power of appeals to benefits for others (Brooks et al., 2020). Reminding the public about the benefits of self-isolation to society can buffer against the negative consequences of quarantine. Specifically, it has been argued that “reinforcing that quarantine is helping to keep others safe, including those particularly vulnerable … can only help to reduce the mental health effect and adherence in those quarantined” (Brooks et al., 2020, p. 919). Apparently, feeling that others will benefit from one’s behavior increases the willingness to endure stressful situations such as self-isolation and makes these situations easier to bear. But do people’s feelings of prosocial responsibility also affect their acceptance of flanking surveillance measures?

In this research, we argue that perceived prosocial responsibility increases both compliance with anti-pandemic measures and support for surveillance, and civil rights and privacy restrictions. Regardless of whether an elevated sense of prosocial responsibility implicitly shifts mental weights from individual to public rights or whether it operates at an affective level that is fueled by the desire to avoid the emotional burden of feeling responsible for others’ suffering, people might feel that the protection of their individual rights matters less than the protection of a common good, such as public health. A sense of prosocial responsibility may act as a blanket measure that heightens a person’s focus on others’ well-being at the expense of tuning down the fight for individual rights. Thus, we predict that people with higher prosocial responsibility both comply more with quarantine measures, and are also more willing to accept radical measures restricting individual rights in general and privacy more specifically.

We tested these predictions with an online study conducted during the COVID-19 pandemic in the US. Specifically, we examined whether prosocial responsibility predicts on the one hand compliance with self-isolation and self-distancing measures, as prior literature suggests, and on the other hand acceptance of digital surveillance and restrictions of individual rights and privacy, as we propose. In addition, we add valuable insights by assessing and controlling for several relevant variables that could also play a role. Specifically, we included variables that address vulnerability to COVID-19 (perceived self-risk, perceived close other-risk, COVID-19 health status, perceived impact on various facets of one’s life, and perceived impact on state), potentially relevant personality traits (narcissism, belief in free will, helplessness, and value of freedom), and demographic variables (age, sex, urban/rural area, and political affiliation).

## Method

### Participants

We recruited 302 US residents online (Prolific). Four participants who failed an attention check (to select a specific answer in one question) were excluded from further analyses. The final sample comprised 298 participants (133 men, 165 women, age 18–80, *M* = 50.71, *SD* = 20.62). A sensitivity power analysis showed that this sample size can reliably detect small to medium effect sizes of ρ = 0.16 (two-tailed) with an alpha level of 0.05 and power of 0.80.

### Procedure

The study was conducted online on May 17, 2020. The following predictor and outcome variables were assessed.

### Predictor Variables

#### Prosocial Responsibility

It was assessed with six items (α = 0.89): “In this COVID-19 pandemic, I feel responsible for the health and life of others,” “In this COVID-19 pandemic, I am doing everything I can to minimize the chances of putting others at risk,” “In this COVID-19 pandemic, I would have a bad conscience if I did something that puts vulnerable people’s health at risk,” “In this COVID-19 pandemic, I feel that my acts have consequences on the lives of others,” “In this COVID-19 pandemic, I would hate it if I did anything that risks vulnerable people’s lives,” and “In this COVID-19 pandemic, not complying with the measures would make me feel almost like a criminal” (1 = *strongly disagree*; 7 = *strongly agree*).

#### Vulnerability to COVID-19

We included several variables that broadly tap vulnerability to the virus. Vulnerability has been shown to be a factor making people susceptible to conformity (Murray and Schaller, 2012; Wu and Chang, 2012) and, thus, might also increase acceptance of restrictions of individual freedoms.

##### Perceived self-risk

It was assessed with four items (α = 0.91): “I consider myself to belong to a high-risk group regarding COVID-1,” “I think I would be severely affected if I am infected with COVID-19,” “I think my life would be at risk if I am infected with COVID-19,” and “In general, I worry about my health with regards to COVID-19” (1 = *strongly disagree*; 7 = *strongly agree*).

##### Perceived close other-risk

It was assessed with four items similar to perceived self-risk (α = 0.94): “I have close others (family, friends, or relatives) who belong to a high-risk group regarding COVID-19,” “Some of my close others (family, friends, or relatives) might be severely affected if they are infected with COVID-19,” “The life of some of my close others (family, friends, or relatives) might be at risk if they are infected with COVID-19,” “In general, I worry about the health of some of my close others (family, friends, or relatives) with regards to COVID-19” (1 = *strongly disagree*; 7 = *strongly agree*).

##### COVID-19 health status

Participants indicated whether they had been tested positive for coronavirus themselves (1 = *yes*; 2 = *no*; 3 = *rather not say*), and the same for any of their close relations (family, close friends).

##### COVID-19 impact on life facets

Participants were asked how negatively or positively the COVID-19 pandemic has affected each one of the following facets of their lives: job, income, emotional well-being, physical well-being, personal relationships (1 = *very negatively*; 7 = *very positively*).

##### COVID-19 impact on state

We measured how badly the state where they had been during lockdown was hit by COVID-19 (1 = *not at all badly*; 7 = *very badly*).

#### Personality Traits

Additionally, we included the following potentially relevant personality traits.

##### Narcissism

Narcissists are self-absorbed and manipulative individuals with a strong sense of specialness and entitlement, a lack of empathy, and a proclivity to exploitation (Thomaes et al., 2018). Therefore, it is reasonable to assume that narcissists should be less likely to comply with measures that stress the protection of others (Grover, 2020, April 18), let alone limit their own freedoms for the common good. Narcissism was assessed with a scale adopted from Webster and Jonason (2013), which comprises four items (α = 0.82; e.g., “I tend to want others to admire me”; 1 = *strongly disagree*; 7 = *strongly agree*).

##### Belief in free will

This is another relevant predictor because it corresponds to a combination of responsibility and autonomy (Nahmias et al., 2005). Believing in free will entails acceptance that individuals are autonomous and responsible and have the capacity to act in different ways in the same situation. Belief in free will was assessed with the free will subscale of the FAD–Plus (Paulhus and Carey, 2011), which comprises seven items (α = 0.85; e.g., “People must take full responsibility for any bad choices they make,” “People have complete free will”; 1 = *strongly disagree*; 7 = *strongly agree*).

##### Helplessness

It refers to the feeling that one has no control over a situation due to repeated experiences with aversive stimuli, which can lead to failure to use opportunities to avoid these stimuli, even when control is possible (Seligman, 1972). Privacy is essentially linked to personal control (Brandimarte et al., 2013). Therefore, people who feel helpless and deprived of personal control might also be less motivated to protect their privacy and safeguard their individual rights, even when they have the opportunity to do so. Helplessness was assessed with the perceived helplessness subscale of the Depressive Attributions Questionnaire (Kleim et al., 2011), which comprises four items (α = 0.86; e.g., “I feel helpless when bad things happen”; 1 = *strongly disagree*; 7 = *strongly agree*).

##### Value of freedom

Individual differences in the value of freedom might also predict the extent to which individuals are willing to sacrifice privacy and individual rights. Participants ranked nine values taken from the Rokeach Value Survey (Rokeach, 1973) into an order of importance to them, as guiding principles in their life. Of interest to this study were the values “Freedom (independence, free choice)” and “National security (protection from attack).” We created a new variable that indicates how much higher freedom is ranked compared to national security by subtracting the freedom rank from the national security rank.

#### Demographic Variables

We collected information about sex, age, area (1 = *rural*; 7 = *urban*), and political affiliation (1 = *democrat*; 7 = *republican*).

### Outcome Variables

#### Compliance With Measures

Compliance with measures against COVID-19 (“To what extent have you been following these measures in the past months?”) was measured with two items in two domains (α = 0.68)^1^ : “Self-isolation (staying home even without having any symptoms)” and “Social distancing (maintaining a safe distance from others)” (1 = *Never*; 2 = *Rarely*; 3 = *Sometimes*; 4 = *About half the time*; 5 = *Frequently*; 6 = *Most of the time*; 7 = *Always*).

#### Willingness to Sacrifice Privacy

It was measured with two items (α = 0.95) following a short explanation that “as a way to deal with the COVID-19 pandemic, several countries have adopted measures that require extensive surveillance (e.g., through collecting data on people’s mobile phones and monitoring their movements)”: “In your opinion, do governments have the right to limit people’s privacy and impose surveillance for the protection of public health?” and “Are you willing to sacrifice your privacy and accept surveillance for the sake of public health?” (1 = *definitely no*; 7 = *definitely yes*).

#### Past Surveillance Acceptance

It was assessed by summing up how many of the following seven actions participants have already done as a way to combat the pandemic (α = 0.58)^2^ : “Install an app on your mobile phone that monitors information about your movements (e.g., where you are going),” “Install an app on your mobile phone that monitors information about your physical contacts (e.g., with whom you are in contact),” “Wear a bracelet that monitors information about your movements (e.g., where you are going),” “Wear a bracelet that monitors information about your physical contacts (e.g., with whom you are in contact),” “Wear a bracelet that monitors information about your health (e.g., your temperature),” “Allow companies (e.g., airlines, your employer) to have access to your medical records,” “Allow companies (e.g., cafes and restaurants, stores) to measure your temperature before entering a venue.”

#### Willingness to Accept Surveillance

It was assessed with seven items (α = 0.92) asking participants to indicate their willingness to accept the same measures as in past surveillance acceptance in the future (“How willing are you to do the following in order to fight against the current pandemic or other similar pandemics in the future?”; 1 = *not willing at all*; 7 = *very willing*).

#### Individual Freedoms Versus Public Health

Participants first read that “in times of crises, leaders and policy-makers sometimes have to take decisions that require a trade-off between individual rights (freedom, autonomy, privacy, self-determination) and public health.” As an example, it was mentioned that “in the current pandemic, world leaders restricted some individual rights for the sake of protecting all citizens’ health.” Then, participants indicated what they would prioritize if such a trade-off were inevitable with a single item (“In your opinion, whenever such a trade-off is inevitable, what should be prioritized, individual freedoms or public health?”; 1 = *definitely individual freedoms*; 6 = *definitely public health*).

## Results

Descriptive statistics and inter-correlations of all variables are presented in Table 1. Inspection of correlation coefficients indicates that prosocial responsibility was positively correlated with compliance with measures to fight COVID-19, *r* = 0.50, *p* < 0.001; willingness to sacrifice privacy, *r* = 0.46, *p* < 0.001; past surveillance acceptance, *r* = 0.11, *p* = 0.059; willingness to accept surveillance, *r* = 0.41, *p* < 0.001; and prioritizing public health over individual freedoms when a trade-off between the two is inevitable, *r* = 0.57, *p* < 0.001.

**TABLE 1:** (A) Descriptive statistics and inter-correlations (part I). (B) Descriptive statistics and inter-correlations (part II).

**(A)**												
	**1**	**2**	**3**	**4**	**5**	**6**	**7**	**8**	**9**	**10**	**11**	**12**
(1) Prosocial responsibility	–											
(2) Compliance	0.50**	–										
(3) Willingness to sacrifice privacy	0.46**	0.31**	–									
(4) Past surveillance acceptance	0.11	–0.03	0.15**	–								
(5) Willingness to accept surveillance	0.41**	0.24**	0.77**	0.17**	–							
(6) Individual freedoms vs. public health	0.57**	0.41**	0.56**	0.08	0.49**	–						
(7) Perceived self-risk	0.42**	0.32**	0.36**	0.01	0.27**	0.43**	–					
(8) Perceived close other-risk	0.43**	0.28**	0.20**	–0.05	0.14*	0.29**	0.43**	–				
(9) Tested positive/self (1 = *yes*)	–0.01	0.03	–0.06	–0.04	–0.04	0.00	0.02	–0.01	–			
(10) Tested positive/other (1 = *yes*)	0.01	0.03	0.04	–0.04	0.02	0.05	0.13*	0.16**	0.16**	–		
(11) Impact/job	–0.01	0.00	0.11	0.00	0.08	–0.02	0.04	–05	–0.09	–0.02	–	
(12) Impact/income	0.01	0.02	0.01	–0.08	0.01	–0.04	–0.07	0.01	–0.06	–10	0.63**	–
(13) Impact/emotional well-being	−0.21**	–0.11	–0.09	–0.05	−0.13*	−0.13*	0.21**	−0.24**	0.01	−0.15*	0.18**	0.14*
(14) Impact/physical well-being	−0.13*	–0.07	–0.09	–0.03	−0.14*	–0.07	−0.15*	−0.19**	0.01	–0.03	0.06	–0.01
(15) Impact/relationships	0.06	0.05	0.09	–0.02	0.04	0.05	0.04	–0.06	0.01	–0.03	0.02	–0.03
(16) Impact/state	0.37**	0.31**	0.27**	0.01	0.23**	0.37**	0.25**	0.24**	0.00	0.16**	−0.13*	–0.08
(17) Narcissism	–0.10	−0.18**	0.04	0.13*	0.05	–0.04	−0.19**	–0.05	0.04	0.01	–0.10	–0.10
(18) Free will	–0.10	–0.05	–0.07	0.12	–0.05	–0.11	–0.08	−0.14*	–0.04	–0.05	0.08	0.04
(19) Helplessness	–0.03	–0.03	–0.04	0.00	–0.01	0.01	−0.18**	0.06	0.01	–0.03	–0.01	–0.05
(20) Value of freedom	−0.21**	−0.19**	−0.23**	−0.13*	−0.23**	−0.23**	−0.29**	−0.17**	0.00	–0.02	–0.03	–0.02
(21) Age	0.11	0.14*	0.22**	–0.03	0.12*	0.08	0.53**	0.07	0.02	0.04	0.21**	0.14**
(22) Sex (1 = *male*)	–0.10	–0.07	–0.01	0.04	–0.04	–0.03	–0.07	–0.07	0.05	0.06	–0.10	–0.09
(23) Area^1^	0.20**	0.17**	0.13*	0.08	0.11	0.21**	0.08	0.12*	–0.06	0.01	0.01	0.05
(24) Political affiliation^2^	−0.32**	−0.23**	−0.28**	0.10	−0.23**	−0.39**	−0.16**	−0.18**	–0.07	–0.10	0.08	0.02
Cronbach’s alpha	0.89	0.68	0.95	0.58	0.92	–	0.91	0.94	–	–	–	–
*M*	5.82	6.23	3.95	0.24	3.38	4.68	4.44	5.93	–	–	3.35	3.39
*SD*	0.98	0.82	2.13	0.66	1.72	1.43	1.72	1.72	–	–	1.12	1.20
Minimum	1.33	2.50	1.00	0.00	1.00	1.00	1.00	1.00	–	–	1.00	1.00
Maximum	7.00	7.00	7.00	6.00	7.00	6.00	7.00	7.00	–	–	7.00	7.00

**(B)**												
	**13**	**14**	**15**	**16**	**17**	**18**	**19**	**20**	**21**	**22**	**23**	**24**

(13) Impact/emotional well-being	–											
(14) Impact/physical well-being	0.45**	–										
(15) Impact/relationships	0.34**	0.22**	–									
(16) Impact/state	−0.15**	–0.02	0.04	–								
(17) Narcissism	–0.08	–0.04	–0.05	–0.02	–							
(18) Free will	0.26**	0.17**	0.12*	–0.05	–0.00	–						
(19) Helplessness	−0.27**	–0.10	−0.18**	0.05	0.30**	−0.24**	–					
(20) Value of freedom	0.10	0.02	0.01	–0.09	0.08	–0.08	0.06	–				
(21) Age	0.10	0.13*	0.16**	0.04	−0.39**	0.03	−0.42**	−0.19**	–			
(22) Sex (1 = *male*)	0.08	0.11	–0.04	0.01	0.11	–0.05	–0.01	0.03	–0.01	–		
(23) Area^1^	–0.11	–0.08	–0.09	0.10	0.04	–0.08	–0.05	–0.00	−0.12*	0.10	–	
(24) Political affiliation^2^	0.14*	0.13*	0.06	−0.22**	0.01	0.33**	–0.01	–0.03	0.05	0.08	−0.16**	–
Cronbach’s alpha	–	–	–	–	0.82	0.85	0.86	–	–	–	–	–
*M*	2.95	3.53	0.388	4.72	3.29	4.87	3.27	2.42	50.71	–	4.58	3.04
*SD*	1.19	1.05	1.15	1.51	1.25	1.03	1.31	2.82	20.62	–	1.98	2.03
Minimum	1.00	1.00	1.00	1.00	1.00	1.14	1.00	–5.00	18	–	1.00	1.00
Maximum	7.00	7.000	7.00	7.00	7.00	7.00	6.75	8.00	80	–	7.00	7.00

*^∗^*p* < 0.05; ^∗∗^*p* < 0.01; ^1^1 = *rural*, 7 = *urban*; ^2^1 = *democrat*, 7 = *republican.**

### Compliance With Measures

We first examined whether a higher sense of prosocial responsibility is associated with higher compliance with self-isolation and social distancing measures after accounting for all control variables in a step-wise linear regression analysis. In the first step, prosocial responsibility served as predictor and compliance with measures as outcome variable. Results showed that prosocial responsibility was a significant predictor of compliance, *B* = 0.42, *SE* = 0.04, β = 0.50, *p* < 0.001. In step two, we entered as control variables all additional predictors listed in Section “Method.” Results showed that prosocial responsibility remained a significant predictor of compliance after controlling for these 18 variables, *B* = 0.29, *SE* = 0.05, β = 0.34, *p* < 0.001 (see detailed results in Table 2). In line with prior research (Brooks et al., 2020), people who feel more responsible toward others were more likely to comply with the measures that have been imposed to combat the pandemic.

**TABLE 2:** Hierarchical regression analyses.

			**Willingness to**		**Past surveillance**		**Willingness to**		**Individual freedoms**	
	**Compliance**		**sacrifice privacy**		**acceptance**		**accept surveillance**		**vs. public health**	
Prosocial responsibility	*0*.42**	*0*. 29**	*1*.00**	*0*. 69**	*0*.*07*	*0*. 13*	*0*.72**	*0*. 54**	*0*.83**	*0*. 51**
Perceived self-risk		*0*.*04*		*0*.*09*		−*0*.*02*		*0*.*06*		*0*. 24**
Perceived close other-risk		*0*.*03*		−*0*.*09*		−*0*.*06*		−*0*.*14*		−*0*.*07*
Tested positive/self^1^		*0*.*37*		−*1*.*58*		−*0*.*15*		−*0*.*75*		−*0*.*20*
Tested positive/other^1^		−*0*.*05*		*0*.*00*		−*0*.*05*		−*0*.*05*		−*0*.*06*
Impact/job		−*0*.*01*		*0*. 29*		*0*.*05*		*0*.*18*		*0*.*02*
Impact/income		*0*.*01*		−*0*.*17*		−*0*.*08*		−*0*.*09*		−*0*.*01*
Impact/emotional well-being		*0*.*02*		*0*.*07*		−*0*.*02*		−*0*.*01*		*0*.*05*
Impact/physical well-being		−*0*.*01*		−*0*.*14*		−*0*.*03*		−*0*.*17*		*0*.*04*
Impact/relationships		*0*.*02*		*0*.*11*		−*0*.*02*		*0*.*05*		*0*.*04*
Impact/state		*0*. 07*		*0*.*14*		*0*.*00*		*0*.*10*		*0*. 11*
Narcissism		−*0*. 09*		*0*. 29**		*0*. 07*		*0*. 18*		*0*.*01*
Free will		*0*.*02*		*0*.*01*		*0*.*06*		*0*.*03*		*0*.*01*
Helplessness		*0*.*04*		*0*.*06*		−*0*.*01*		*0*.*02*		*0*.*06*
Value of freedom		−*0*.*02*		−*0*. 09*		−*0*.*03*		−*0*. 09**		−*0*. 06*
Age		*0*.*00*		*0*. 02**		*0*.*00*		*0*.*01*		−*0*.*01*
Sex^2^		−*0*.*02*		*0*.*18*		*0*.*04*		*0*.*05*		*0*.*11*
Area^3^		*0*.*03*		*0*.*04*		*0*.*03*		*0*.*02*		*0*.*05*
Political affiliation^4^		−*0*.*03*		−*0*. 19**		*0*.*04*		−*0*. 11*		−*0*. 16**
Multiple *R*	*0*.*50*	*0*.*56*	*0*.*46*	*0*.*60*	*0*.*41*	*0*.*32*	*0*.*41*	*0*.*51*	*0*.*57*	*0*.*68*
Adjusted *R*^2^	*0*.*25*	*0*.*26*	*0*.*21*	*0*.*31*	*0*.*17*	*0*.*04*	*0*.*17*	*0*.*21*	*0*.*33*	*0*.*43*

*Unstandardized coefficients are provided. ^∗^*p* < 0.05; ^∗∗^*p* < 0.01; ^1^Dummy coded (1 = *yes*); ^2^Dummy coded (1 = *male*); ^3^1 = *rural*, 7 = *urban*; ^4^1 = *democrat*, 7 = *republican.**

### Willingness to Sacrifice Privacy

We then tested whether a higher sense of prosocial responsibility is associated also with a higher willingness to sacrifice privacy for the sake of public health. Results showed that prosocial responsibility was a significant predictor of willingness to sacrifice privacy, *B* = 0.10, *SE* = 0.11, β = 0.46, *p* < 0.001. Moreover, prosocial responsibility remained a significant predictor of willingness to sacrifice privacy after entering all control variables, *B* = 0.69, *SE* = 0.13, β = 0.32, *p* < 0.001 (see detailed results in Table 2). Therefore, people higher in prosocial responsibility were more willing to sacrifice their privacy for the sake of public health.

### Past Surveillance Acceptance

Another linear regression showed that prosocial responsibility was a marginally significant predictor of past surveillance acceptance, *B* = 0.07, *SE* = 0.04, β = 0.11, *p* = 0.059. After controlling for the same variables as above, prosocial responsibility became a significant predictor of past surveillance acceptance, *B* = 0.13, *SE* = 0.05, β = 0.19, *p* = 0.010 (see detailed results in Table 2). Therefore, people who feel more responsible toward others in the pandemic have already accepted more surveillance measures.

### Willingness to Accept Surveillance

Results of a linear regression analysis indicated that prosocial responsibility also predicted willingness to accept surveillance in the future, *B* = 0.72, *SE* = 0.09, β = 0.41, *p* < 0.001. The effect of prosocial responsibility on willingness to accept surveillance remained significant after entering the control variables, *B* = 0.54, *SE* = 0.12, β = 0.31, *p* < 0.001 (see detailed results in Table 2). Thus, prosocial responsibility did not only predict past surveillance acceptance but also willingness to accept surveillance in the future.

### Individual Freedoms Versus Public Health

We conducted another regression with prosocial responsibility as predictor and the dilemma between individual freedoms and public health as outcome variable. Results showed that prosocial responsibility was significantly associated with a preference for public health over individual freedoms, *B* = 0.83, *SE* = 0.07, β = 0.57, *p* < 0.001. This association remained significant after controlling for the same variables as before, *B* = 0.51, *SE* = 0.08, β = 0.35, *p* < 0.001 (see detailed results in Table 2). That is, the stronger a person’s sense of prosocial responsibility, the more likely that person prioritizes public health over individual freedoms.

## Discussion

During the COVID-19 pandemic, governments around the world emphasized responsibility toward others as a way to enforce self-isolation and social distancing. In line with a recent review of the literature, which advises public health officials to emphatically communicate the benefits of self-isolation for others (Brooks et al., 2020), we found that a stronger sense of prosocial responsibility predicted compliance with self-isolation and social distancing measures. At the same time, our findings suggest that prosocial responsibility is also associated with acceptance of restrictions of privacy and individual rights. Apparently, feeling responsible for others leads people to devalue their own rights.

Critically, this holds over and above a host of alternative explanations and related variables, such as how much they believe that they personally or their close others are at risk, how much they value freedom, or how negatively various facets of their lives have been affected by the pandemic. This finding implies that prosocial responsibility can be a double-edged sword. On the one hand, it enhances compliance with self-isolation and social distancing, which is of paramount importance in pandemic crises. On the other hand, prosocial responsibility might constitute a Trojan horse for privacy undercuts because it makes people generally accept a loss of individual rights. This finding echoes growing concerns about the potential misuse of digital surveillance methods during the pandemic (e.g., Abbas et al., 2020; Calvo et al., 2020; Roth et al., 2020) and highlights a potential long-term side-effect that may eventually turn out detrimental for all individuals.

Our research contributes to the literature on the effectiveness of prosocial appeals more broadly (e.g., Small and Cryder, 2016; Thornton et al., 2019), by highlighting the role of prosocial responsibility in the fight against a pandemic (Brooks et al., 2020). Moreover, our findings contribute to the privacy literature. Thus far, the privacy literature has focused on the individual when examining predictors of privacy behavior, such as desire for control over personal information (Phelps et al., 2001), knowledge about risks (Park et al., 2012), and privacy concerns (Gerber et al., 2018). Our research adds a novel social dimension to recent research, which has begun to investigate the interdependent aspects of privacy (Kamleitner and Mitchell, 2019). In many situations, individuals endanger others’ privacy for their self-interest (e.g., when allowing apps access to their contacts). Here, we show the opposite. Out of concern about others, individuals might endanger their own privacy. Both studies underscore the role of social context in people’s privacy-related behaviors and point out the need for more research in this direction.

Besides the crucial role of prosocial responsibility, the current research provides insights into the role of other variables in the pandemic. In terms of COVID-19-related variables, we found that perceived vulnerability in its various forms (perceived self-risk or close other-risk, age, COVID-19 impact on state) was consistently associated with both higher compliance with the measures against COVID-19 and higher acceptance of surveillance and privacy restrictions, converging with prior research showing that vulnerability increases conformity (Murray and Schaller, 2012; Wu and Chang, 2012). In terms of demographic variables, compliance with measures as well as acceptance of surveillance and privacy restrictions were higher among democrats (vs. republicans) and among people living in urban (vs. rural) areas.

In terms of personality traits, we found that narcissism was associated with lower compliance, confirming the assumption that in this situation, too, narcissists might indeed behave selfishly and disregard the consequences of their behavior on others (Grover, 2020, April 18). Moreover, a higher belief in free will was marginally associated with lower prosocial responsibility and lower prioritization of public health vis-a-vis individual freedoms. Extending prior findings that belief in free will is associated with a more punitive attitude toward wrongdoers (Baumeister and Brewer, 2012), our findings suggest that belief in free will might also imply that everyone is responsible only for themselves and not for others. A higher value of freedom was also associated with lower acceptance of privacy restrictions. However, contrary to predictions, feeling helpless was unrelated with the willingness to make sacrifices in one’s privacy or accept surveillance.

By investigating and controlling for a range of relevant predictors of people’s willingness to accept a loss of individual rights, our research adds several novel but preliminary insights to the study of this timely phenomenon. Future research should follow up on the multiple leads this initial exploration provides. Most importantly, our research is the first to demonstrate a robust link between people’s sense of prosocial responsibility and their willingness to sacrifice individual rights, in particular privacy. Future research is needed to corroborate this link in other cultural contexts and with measures that are not dependent on self-reports. Should results be as robust as we expect, then the prosocial appeals used to fight the pandemic might come at a potential long-term price to individual rights.

## Data Availability Statement

The raw data supporting the conclusions of this article will be made available by the authors, without undue reservation.

## Ethics Statement

Ethical review and approval was not required for the study on human participants in accordance with the local legislation and institutional requirements. The patients/participants provided their written informed consent to participate in this study.

## Author Contributions

MK conducted the study and analyzed the data in consultation with BK. MK wrote the manuscript. BK reviewed and edited the manuscript. Both authors conceptualized and designed the study and contributed to the article and approved the submitted version.

## Conflict of Interest

The authors declare that the research was conducted in the absence of any commercial or financial relationships that could be construed as a potential conflict of interest.

## Footnotes

1 We collapsed the two items that comprise this measure for the sake of parsimony, even though internal consistency for this measure is at the lower end of acceptable values (Nunnally and Bernstein, 1994). Self-isolation and social distancing were moderately correlated with each other, *r* = 0.54, *p* < 0.001, probably because they differ in difficulty (self-isolation might be considered harder than social distancing), and therefore participants might be practicing one more than the other.

2 Low internal consistency of this measure can be largely attributed to the fact that the majority of participants (82.6%) had thus far accepted only a few of these measures – probably because many of these are not yet widely implemented.
